# Characterization of ferrite nanoparticles for preparation of biocomposites

**DOI:** 10.3762/bjnano.8.127

**Published:** 2017-06-13

**Authors:** Urszula Klekotka, Magdalena Rogowska, Dariusz Satuła, Beata Kalska-Szostko

**Affiliations:** 1 Institute of Chemistry, University of Bialystok, Ciołkowskiego 1K, 15-245 Bialystok, Poland; 2 Faculty of Physics, University of Bialystok, Ciołkowskiego 1L, 15-245 Bialystok, Poland

**Keywords:** albumin, EDX, glucose oxidase, IR spectroscopy, lipase, magnetic nanoparticles, surface immobilization, trypsin, X-ray diffraction

## Abstract

Ferrite nanoparticles with nominal composition Me_0.5_Fe_2.5_O_4_ (Me = Co, Fe, Ni or Mn) have been successfully prepared by the wet chemical method. The obtained particles have a mean diameter of 11–16 ± 2 nm and were modified to improve their magnetic properties and chemical activity. The surface of the pristine nanoparticles was functionalized afterwards with –COOH and –NH_2_ groups to obtain a bioactive layer. To achieve our goal, two different modification approaches were realized. In the first one, glutaraldehyde was attached to the nanoparticles as a linker. In the second one, direct bonding of such nanoparticles with a bioparticle was studied. In subsequent steps, the nanoparticles were immobilized with enzymes such as albumin, glucose oxidase, lipase and trypsin as a test bioparticles. The characterization of the nanoparticles was acheived by transmission electron microscopy, X-ray diffraction, energy dispersive X-ray and Mössbauer spectroscopy. The effect of the obtained biocomposites was monitored by Fourier transform infrared spectroscopy. The obtained results show that in some cases the use of glutaraldehyde was crucial (albumin).

## Introduction

Nanoparticles are important ingredients in the fabrication of biocomposites, and therefore, the surface functionalization of nanoparticles attracts great interest among scientists [1–2]. Tests that aim at the functionalization of nanoparticles with organic compounds are becoming the most popular due to the wide potential applications of such hierarchical structures. On demand surface characteristics allow further immobilization of proper biological structures [3], and as a result, biocomposites are obtained. Metallic nanoparticles might be directly combined with organic compounds or via complicated linkers in the form of organized monolayers on the nanoparticles surface [4] or rather as random structures. Such a functional type of monolayer can be used for further modification by covalent or noncovalent bonding with a third set of particles. In particular, noncovalent interactions are the most important in terms of biological aspects [5]. A drawback of applying magnetic nanoparticles is that they possess a strong tendency to agglomerate due to not only van der Waals or electrostatic forces but also magnetic interactions [6]. This explains why the stabilization of nanoparticles with active surface compounds is so important [7]. Well-determined or highly engineered surface modification can expand the usability of spontaneous features of nanomaterials (oxidation state, affinity to special compounds, etc.). Functionalization can ensure connection of the nanoparticle surface self-assembled layers with free active bonds [8].

The integration of nanostructures with biomolecules leads to the fabrication of a novel hybrid system that couples recognition or catalytic properties of biomaterials with attractive electronic, optical, magnetic and structural characteristics of specific nanoparticles in one hierarchical structure [9]. In such systems, nanoparticles can be functionalized with various biomolecules through different linkage chemistry [10–12].

Nanocomposites based on magnetic nanoparticles have a huge advantage over nonmagnetic nanopartices due to the synergy of magnetic properties of the core particles with surface bioactivity or biomolecule recognition. The described hybrid system possesses very useful magnetic properties, which can be tunable and used as manipulation tools and, at the same time, interaction with living cells can be obtained. The use of an external magnetic field quite often helps in removing toxins from healthy cells [13–14] via modified nanoparticles having a sieve-like property.

The main aim of this work is to study immobilization effects of biological particles to the selected ferrite nanoparticles. Detailed studies in this subject on particular systems provide conclusions and give ideas as to how to apply magnetic nanoparticles in the fields of medical, biological or environment protection. Nevertheless, precise studies on the physicochemical properties of the ferrite core is of main importance while its application is considered. Therefore, in this paper, we selected four types of ferrite nanoparticles (magnetite, and magnetite doped with Ni, Co, or Mn elements, respectively), and studies on the immobilization of biologically active particles were done. For this purpose, we have used nanoparticles with or without attached glutaraldehyde that served as a linker between the nanoparticle and enzymes and gives more space for interaction. The enzymes tested in this paper were: albumin, glucose oxidase, lipase, and trypsin. This study is a continuation of our previous papers, where core–shell ferrite nanoparticles were tested in similar manner [15–16].

## Results and Discussion

### Characterization of ferrite nanoparticles

#### Transmission electron microscopy (TEM)

**Figure 1:**
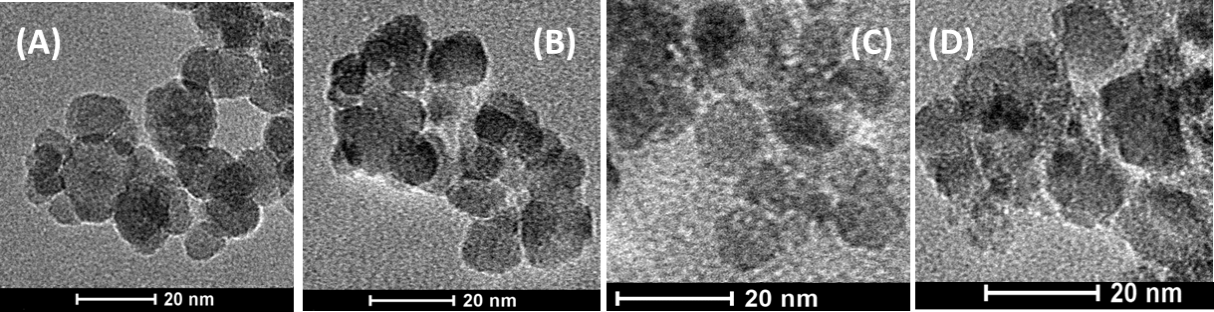
TEM images of the ferrite nanoparticles: A) Fe_3_O_4_, B) Co_0.5_Fe_2.5_O_4_, C) Mn_0.5_Fe_2.5_O_4_, D) Ni_0.5_Fe_2.5_O_4_.

Each type of ferrite nanoparticle studied was imaged by TEM. The resulting pictures are collected in Figure 1.

The presented TEM images of the prepared series of ferrite nanoparticles show that, in general, the particles have a well-defined, round structure. The examined ferrites differ, however slightly, in the calculated average size (diameter). The analysis of the presented images shows that the mean diameter of the observed objects varies from 12 ± 3 nm to 16 ± 2 nm (Table 1). Moreover, the presented nanoparticles have a strong tendency to agglomerate due to their significant magnetic interaction that competes with the much weaker electrostatic repulsion. The interplay between these two facts is expected due to the nature of ferrites and difficulties in obtaining a sufficient surface coverage by surfactants. On the other hand, the preparation of the samples for TEM actually disturbs the functionality of primarily used surfactants, which can no longer maintain the separation of the particles. The lack of self-assembly also causes worse particle separation, as seen in the TEM images. It is also observed that after modification of the inorganic core, the size distribution increases in comparison to Fe_3_O_4_ nanoparticles (Table 1), while the average size of the ferrite core decreases. This suggests that Co, Mn, and Ni ions influence the crystallization process (which turned out to be slower in comparison to Fe). This observation explains the observed differences in the Mössbauer spectra from those presented in previous studies [17].

**Table 1:** Diameter of nanoparticles determined from TEM images and estimated average grain diameters, unit cell parameters and strain values from the most intense XRD patterns.

Nanoparticle composition	Particle diameter [nm] TEM	Particle diameter [nm] XRD	Cell parameter [Å] ± 0.02 XRD	Strain [10^−3^] ± 0.2 XRD

Fe_3_O_4_	16 ± 2	17 ± 1	8.34	4.8
Co_0.5_Fe_2.5_O_4_	12 ± 3	12 ± 2	8.35	4.9
Mn_0.5_Fe_2.5_O_4_	13 ± 3	11 ± 2	8.39	4.8
Ni_0.5_Fe_2.5_O_4_	12 ± 3	9 ± 2	8.34	4.4

#### Energy dispersive X-ray analysis (EDX)

**Figure 2:**
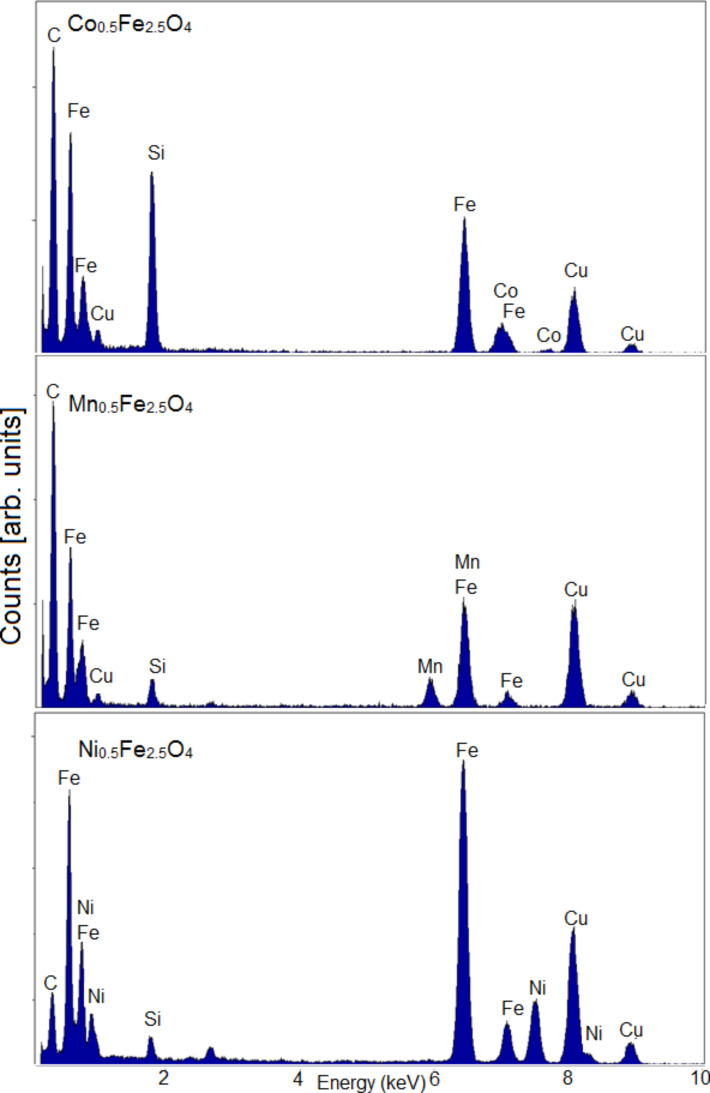
EDX spectra of the studied ferrite nanoparticles.

To confirm the substitution of Fe by other 3d elements, EDX analysis was performed. For this purpose, the particles analyzed previously with TEM were used. The assumed molar ratio of Fe/Me was confirmed to be 2.5:0.5. Element specific line scans of ferrite nanoparticles show the presence of all substituted metals in the studied particles. This demonstrates the successful modification of the ferrite nanoparticle core. The obtained results clearly prove the ferrite nanoparticle compositions and therefore illustrate the easy modification of the chemical composition of the core. In Figure 2 and Table 2, the EDX spectrum and the elemental compositions are collected.

**Table 2:** Elemental composition of the tested ferrite nanoparticles.

Element	Weight [%]		
	Co_0.5_Fe_2.5_O_4_	Mn_0.5_Fe_2.5_O_4_	Ni_0.5_Fe_2.5_O_4_

Fe	82 ± 4	82 ± 4	87 ± 4
Co	18 ± 3	–	–
Mn	–	18 ± 4	–
Ni	–	–	13 ± 5

#### X-ray diffraction (XRD)

**Figure 3:**
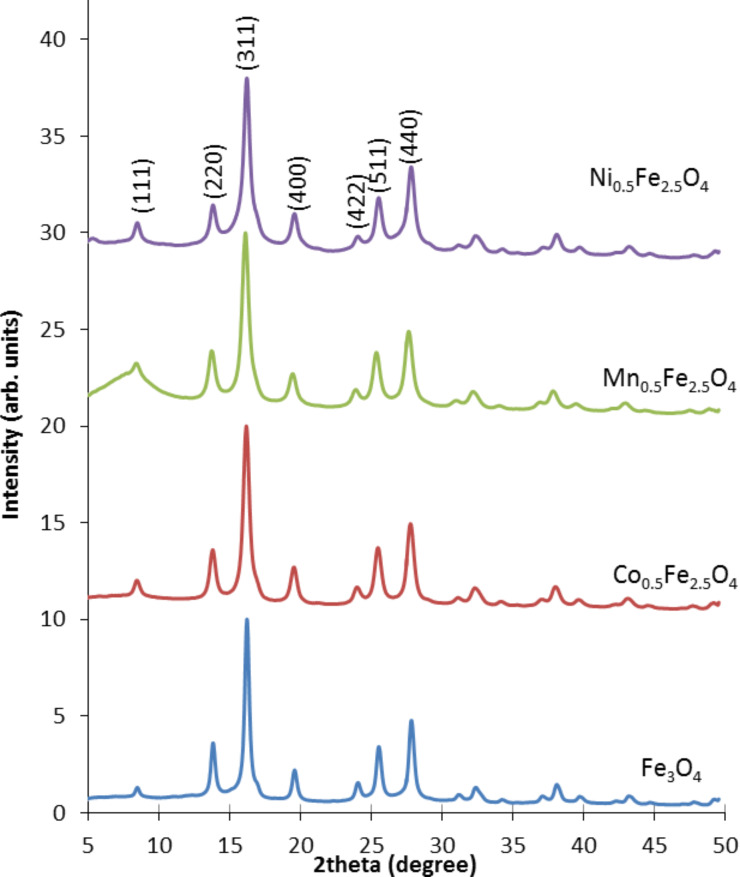
X-ray spectra of ferrite nanoparticles.

All resultant ferrite nanoparticles were measured by X-ray diffraction to see if any changes appear in the crystal structure of the nanoparticles after composition modification. The obtained diffractograms are presented in Figure 3. From a previous work [17], it was expected that the crystal structure of magnetite/maghemite would remain unchanged and the Me ions would relocate rather randomly in the Fe crystallographic positions. There are no traces of any crystalline separation which would be observed as extra diffraction peaks. Therefore, it is concluded that Me ions are likely incorporated into Fe sites. This scenario is also expected after the qualitative observation of the increase of the average line width of the XRD patterns collected for the studied materials [18]. It is clear that after the substitution of Fe by Me, these lines become wider in comparison to pure magnetite (see Figure 3). The reason why the line width has changed is also due to the combination of few significant contributions: variable composition, local stress, modification of the cell size, etc. [19–20]. All these influences shape the diffractograms in an important way, and thereby the value of the calculated average particle size (which appears smaller than it is in reality) [21]. But even so, the correlation between TEM and XRD is satisfactory.

The X-ray spectra presented in Figure 3 show that hkl indexes in every spectra are typical for magnetite/maghemite structure (111) (220) (311) (222) (400) (422) (511) (440) [16,22]. Qualitatively it can be seen that the signal width increases for the nanoparticles with embedded Co, Mn or Ni elements in the crystalline structure in comparison to pure magnetite, while the *x* axis positions remain the same [23]. Therefore, the XRD data indicates the preservation of the structure of magnetite regardless of substitution of other ions (Co, Ni, and Mn) in the crystallographic positions of Fe, and no preference in occupation is seen.

The average grain size of the crystallites was calculated from the X-ray spectra using Scherrer’s equation (Equation 1) [24]:

[1]
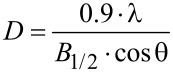


where *D* – grain size [Å], λ – wavelength (for Mo source it is 0.7136 Å), *B*_1/2_ – full width at half maximum intensity of the peak [rad], and θ – diffraction angle [rad]. The obtained results are presented in Table 1.

The average grain sizes of the nanoparticles with Co, Mn and Ni dopant elements calculated from Equation 1 differ from the pure magnetite crystalline grain size (Table 1). This modification/decrease is due to the change/increase of the width of the structural peaks, which is a consequence of particle composition, particle size, stress and many other important factors previously mentioned [25]. The obtained average particle size was found to be within the error bars for both the XRD and TEM particle size analysis. A decreasing trend is also preserved. This is all reflected in the strain value which is rather high but depends on the synthesis conditions. The parameters calculated from the XRD unit cell are very close to that expected for bulk magnetite (for details see Table 1) [24].

#### Mössbauer spectroscopy (MS)

**Figure 4:**
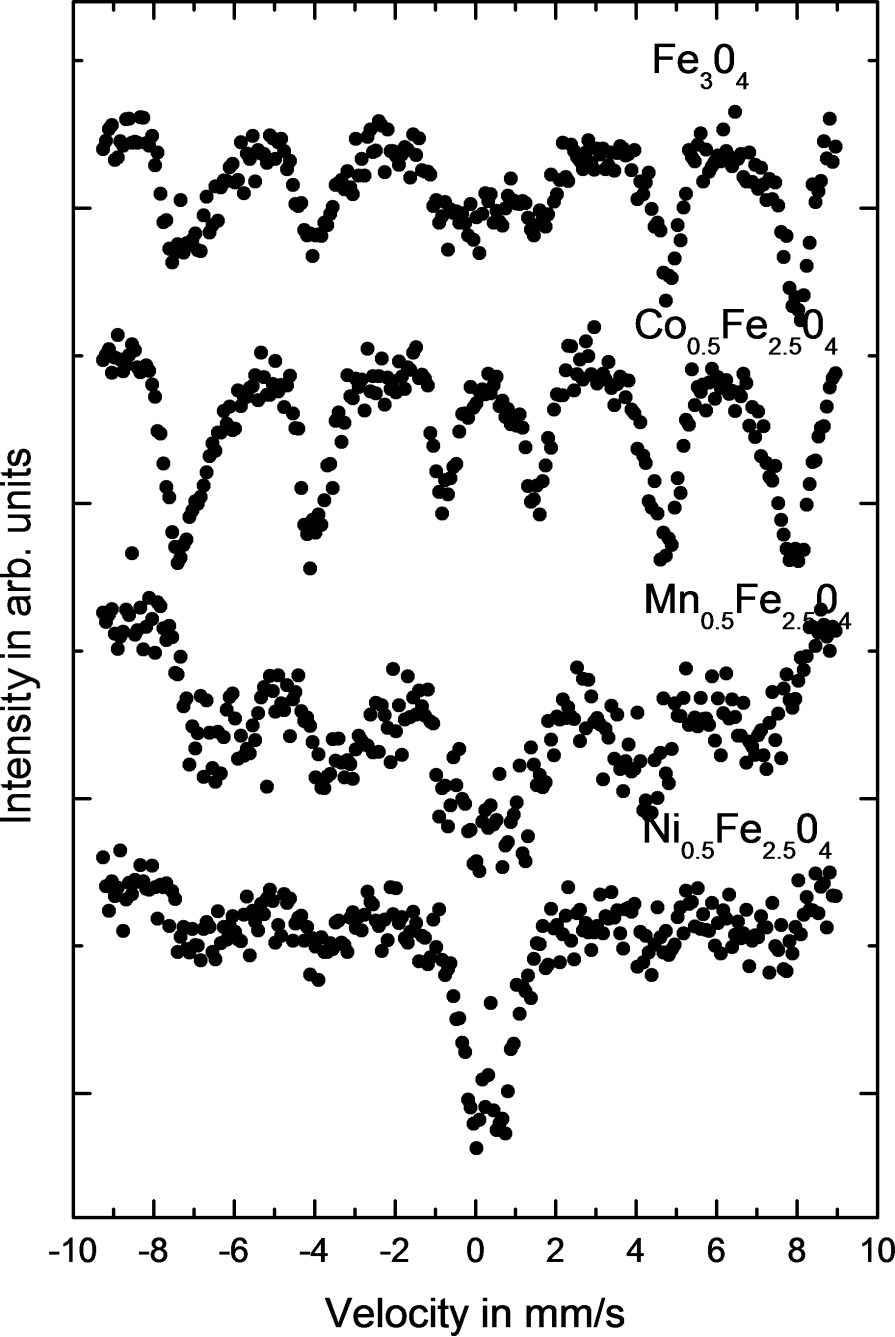
Mössbauer spectra of various ferrite nanoparticles.

The magnetic characterization of the ferrite core was performed by Mössbauer spectroscopy. A standard spectrometer working in constant acceleration mode at RT was used. The results of this study are depicted in Figure 4.

A qualitative analysis of the Mössbauer spectra presented in Figure 4 allows us to conclude that the magnetic characteristics of the presented nanoparticles are not the same at RT and the spectra are strongly dependent on the doping material. At RT, magnetite and Co_0.5_Fe_2.5_O_4_ are almost totally below the superparamagnetic blocking temperature. Especially in case of Co it is seen that even when the average particle size decreases, the mean hyperfine field increases. This phenomenon can only be caused by the influence of Co on Fe in a way that the Fe magnetic moment increases when Co is in the nearest surrounding of the Fe nuclei. Such a scenario is in good agreement with the observation in other systems [26]. Particles substituted with Mn are very close to superparamagnetic *T*_B_, which is estimated to occur when the contribution from sextets and doublets reaches equilibrium (50%–50%). On the contrary, Ni causes a significant increase of *T*_B_ in comparison to Fe_3_O_4_. This observation, however, should be also combined with the result that the average particle size also decreases for Ni_0.5_Fe_2.5_O_4_ in comparison to magnetite and Co_0.5_Fe_2.5_O_4_. The conclusion of which phenomena plays the more prominent role is not possible at this stage of the investigation and requires more studies, especially at low temperature [27]. This result, however, is in good agreement with data published previously [18]. Detailed studies on Co, Mn and Ni doping and its influence on RT properties as observed by Mössbauer spectroscopy can be found in [17].

#### Nanoparticle–enzyme biocomposite characterization

The studied nanoparticles were divided into two groups, one was firstly modified with glutaraldehyde (which served as an extra linker between the nanoparticle and enzyme) and then respective enzymes were immobilized. For the second group, enzymes were attached without any previous surface modification. The prepared composites were measured by FTIR spectroscopy after drying.

#### Infrared spectroscopy

**Figure 5:**
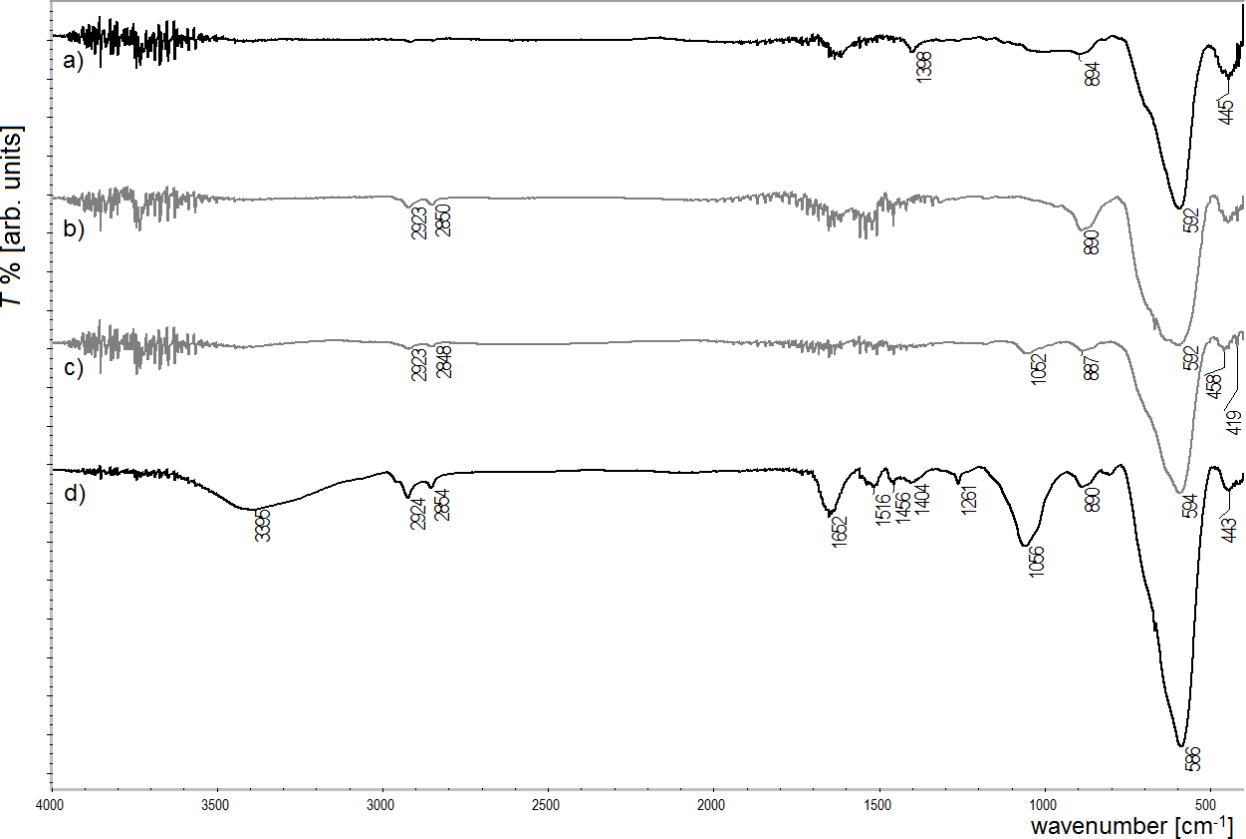
IR spectra of a) Fe_3_O_4_; b) Fe_3_O_4_ after surface phase change; c) Fe_3_O_4_ after modification with glutaraldehyde; d) Fe_3_O_4_ after attachment of trypsin.

**Figure 6:**
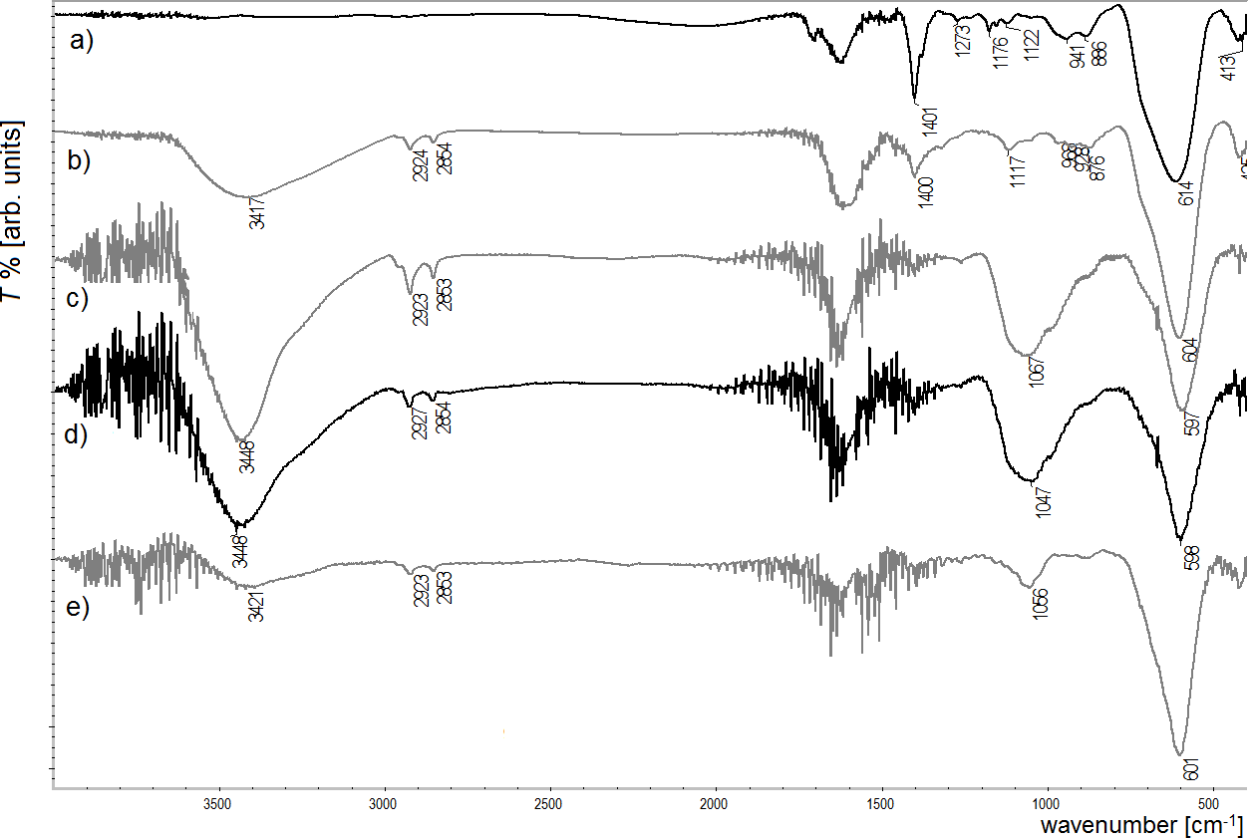
IR spectra of biocomposites with Ni_0.5_F_2.5_O_4_ nanoparticles in the core and with different enzymes on the surface: a) reference sample without modification, b) albumin, c) glucose oxidase, d) lipase, e) trypsin.

**Figure 7:**
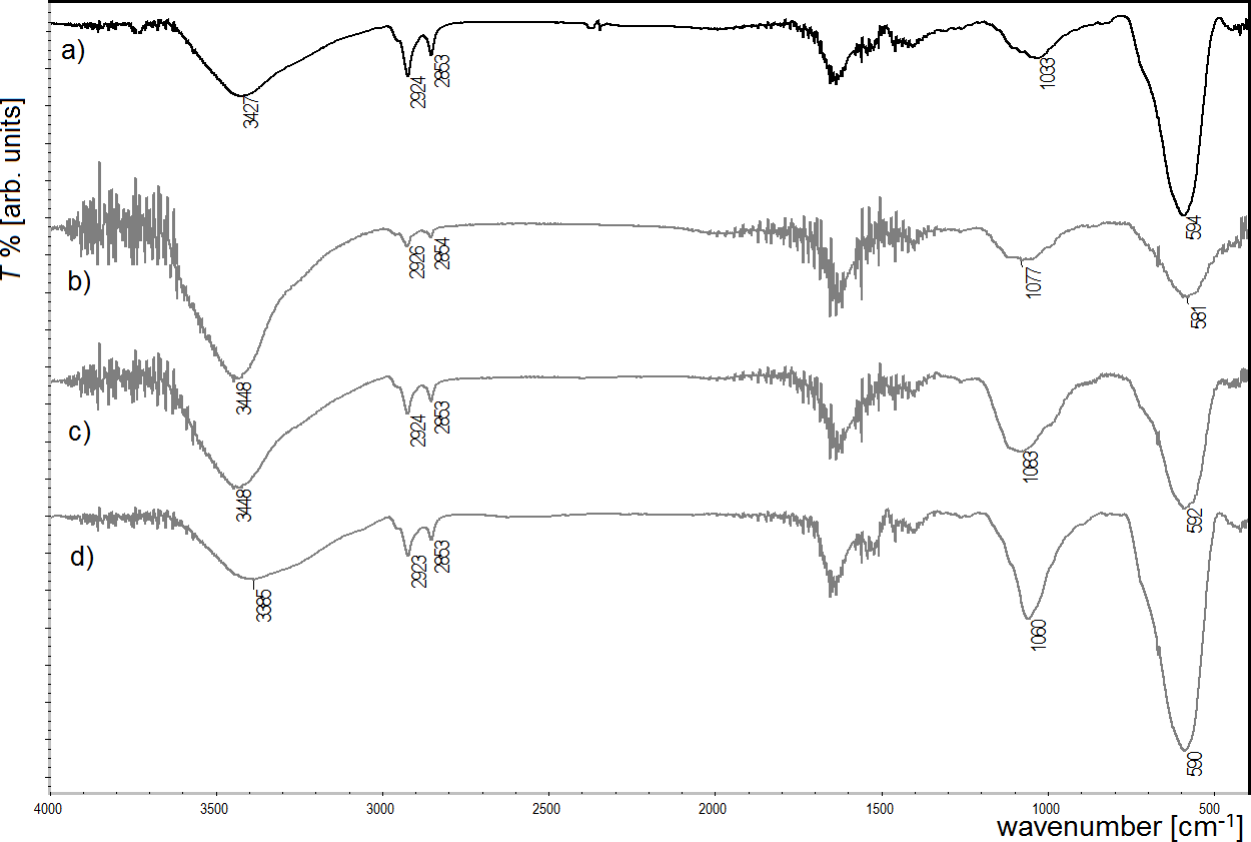
IR spectra of biocomposites with Mn_0.5_Fe_2.5_O_4_ nanoparticles modified by glutaraldehyde and different enzymes: a) albumin, b) glucose oxidase, c) lipase, d) trypsin.

Selected ferrite nanoparticle samples were tested by IR spectroscopy to observe changes taking place on the nanoparticle surface after every step of the biocomposite fabrication. The resulting spectra are presented in Figures 5–7. To limit the number of plots, the selection of IR data was done due to the fact that all changes have the same character in all cases for each ferrite core and surface modification.

The IR spectra presented in Figures 5–7 show changes on the nanoparticle surface after every modification step. The spectra of the magnetite nanoparticles (Figure 5) show bands at 592 cm^−1^ which originate from the Fe–O bond typical for magnetite [28–29]. The small signals appearing at 445 cm^−1^ are related to the Fe–O bond from hematite and the band at 894 cm^−1^ from the O–H bonds in goethite [30], which suggests a weak surface oxidation process. The signals at 1398–1630 cm^−1^ are present due to –CH_2_ binding in TBAOH. After surface modification (spectra b), new bands occur at 1452 cm^−1^ and 2850–2923 cm^−1^ caused by the presence of –NH_2_ and –COOH bonds, respectively [31]. This observation proves the successful surface modification of the nanoparticles. The functionalization of magnetite with glutaraldehyde (spectra c) results in the presence of a new signal at 1052 cm^−1^ that originates from –CH=O in this compound [32]. The last spectra (d) were collected after the attachment of trypsin, and here a small signal at 1056 cm^−1^ occurs, which suggests the presence of the tested enzyme in the sample. Similar changes in the IR spectra were observed previously [15–16] and they were explained to be the result of surface modification.

In Figure 6, the IR spectra of the biocomposites combined with surface-modified NiFe_2_O_4_, and four tested enzymes are presented. In the cases where glucose oxidase, lipase and trypsin were attached, a strong signal at around 1050 cm^−1^ can be seen, which can be seen as the successful bonding of these enzymes. Nevertheless, spectra with albumin do not present such a band, which implies the lack of this enzyme on the nanoparticle surface.

The last spectra (Figure 7) shows biocomposites with Mn_0.5_Fe_2.5_O_4_ modified with glutaraldehyde and the four tested enzymes. Here, in comparison to Figure 7B, all tested enzymes were successfully attached to the nanoparticles. This study demonstrates that for some enzymes (like albumin) the application of a linker (such as glutaraldehyde) is necessary to obtain a desired hybrid structure. This can be caused by structural differences between the studied bioparticles.

## Conclusion

It is shown that the presented magnetic nanoparticles can be used as a transfer medium for bioactive particles. The modification of the core structure can modify the magnetic response of the particle to the external magnetic field while the size of the particles is a critical parameter. It was observed that superparamagnetic fluctuations are blocked (or not blocked) at RT for nanoparticles with a diameter smaller than the reference magnetite due to elemental substitution. Therefore, at any stage of the fabrication procedure, additional characteristics can be supplemented (e.g., ferrite core differentiation or surface modification). The improvement of the surface functionalization by introduction of active linkers is, in some cases, a crucial issue as it was presented in the case of albumin. This paper is a follow up on the studies carried out on the physico-chemical characterization of ferrite cores and shows importance in the application in bio-related systems.

## Experimental

### Materials and apparatus

For the synthesis of ferrite nanoparticles, the following chemicals were purchased from POCH: FeCl_3_·6H_2_O, FeCl_2_·4H_2_O, NiCl_2_·6H_2_O, CoCl_2_, MnCl_2_, NH_3,_ acetone, and tetrabutylammonium hydroxide (TBAOH) was purchased from Sigma-Aldrich. To modify the surface of ferrite nanoparticles, toluene (POCH), oleic acid (POCH) and oleylamine (Sigma-Aldrich) were used. For the functionalization of nanoparticles by glutaraldehyde, trizma hydrochloride (Tris-HCl) and glutaraldehyde (25% in water) were obtained from Sigma-Aldrich. Four enzymes were attached to the nanoparticles: albumin (from Bovine serum), glucose oxidase (from Aspergillus Niger, type II), lipase (from Porcine Pancreas, type II) and trypsin, in the presence of Tris-HCl or phosphate-buffered saline (PBS), all purchased from Sigma-Aldrich.

The quality of the ferrite nanoparticle cores was analyzed by transmission electron microscope (TEM) on a Tecnai G2 X-TWIN type from FEI. For such purposes, a drop of diluted in ethanol particles was drop-casted on a Cu grid covered with an amorphous carbon film to provide good support for the particles. Energy dispersive X-ray spectra (EDX) were collected during the TEM measurements. The analysis of the crystal structure was done by X-ray diffraction (XRD) on an Agilent Technologies SuperNova diffractometer with a Mo micro-focused source (Kα_2_ = 0.713067 Å). For structural characterization, the nanoparticle powder was placed onto a nylon loop fixed to a proper pin with the help of a high viscous synthetic oil. The FTIR spectra were collected in reflection mode on a Nicolet Magna IR 550 Series II spectrometer in the spectral range 500–4000 cm^−1^. Room temperature Mössbauer spectra were obtained using the spectrometer working in constant acceleration mode with a ^57^Co(Cr) radioactive source.

### Synthesis of ferrite nanoparticles

In the presented paper, magnetite (Fe_3_O_4_) and ferrite nanoparticles containing Co (Co_0.5_Fe_2.5_O_4_), Mn (Mn_0.5_Fe_2.5_O_4_) and Ni (Ni_0.5_Fe_2.5_O_4_) were prepared. The synthesis of these materials was done by the modification of Massart’s method [33]. This is based on the co-precipitation of (0.81 g) Fe(III) and (0.29 g) Fe(II) chlorides in 0.5% ammonia aqueous solution at a temperature of 80 °C under Ar atmosphere [15]. To obtain ferrite with Co, Mn or Ni, half of the Fe(II) salt was replaced by proper Me(II) salt [18]. Finally, the nanoparticles were separated from the solution by the application of the permanent hand magnet, washed in deoxygenated acetone and dried to powder form with a vacuum evaporator. Such material was the basis for further characterization.

### Surface modification and glutaraldehyde functionalization

A prerequisite for successful biocomposite preparation is the surface modification of the obtained nanoparticles with the most useful –COOH and –NH_2_ groups. In such a case, the nanoparticles in powder form were introduced into the mixture of oleic acid, oleylamine, toluene and water in the molar ratio 36:36:18:10. The nanoparticles were mixed and sonicated and then left for four days. As a result, the surface characteristics of the nanoparticles change from aqueous to organic. At the end, the nanoparticles were washed and dried at RT to powder form.

The functionalization of the nanoparticles with glutaraldehyde starts with washing and mixing by sonication of surface-modified nanoparticles in Tris-HCl solution (pH 7.4). Then, a 5% solution of glutaraldehyde was added and the mixture was mixed for 3 h at RT [34]. After that, the nanoparticles were once again washed with Tris-HCl solution and dried at RT overnight.

### Attachment of the tested enzymes

**Figure 8:**
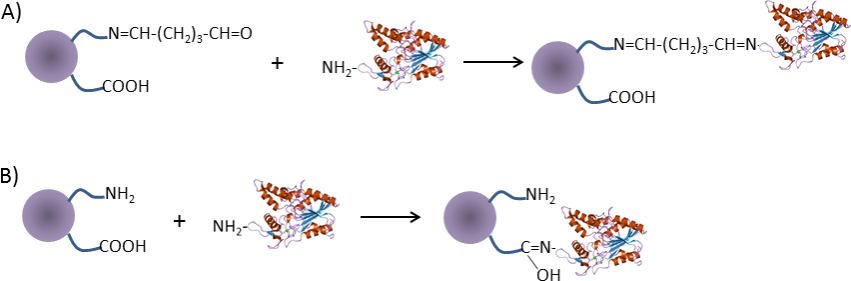
Schematic presentation of two types of biocomposite preparation: A) nanoparticles with glutaraldehyde and enzyme; B) nanoparticles after surface modification and enzyme attachment.

In the present studies, enzymes were attached to the nanoparticles in two ways. In the first one, via glutaraldehyde, and in the second, without it, directly to the –COOH obtained via surface modification with oleic acid, which is schematically presented in Figure 8. Below, we present the methods for attachment of the various enzymes.

1. Albumin: Nanoparticles were washed with Tris-HCl solution, then the mixture of albumin (17 g/L) and Tris-HCl was added to the nanoparticles. The sample was mixed for 2 h in RT and after that washed 3 times with Tris-HCl and dried at RT [35].
2. Glucose oxidase: At first, the nanoparticles were washed with PBS solution, then the mixture of glucose oxidase (5.4 g/L) in PBS solution was added. The whole combination was mixed for 2 h at RT, then washed 3 times with PBS and dried at RT [16,36].
3. Lipase: Attachment of lipase starts with washing the nanoparticles with PBS solution, then the mixture of lipase (14 g/L) and PBS was added to the nanoparticles. The combination was mixed for 2 h in a warm water bath. At the end, the obtained composite was washed with PBS and dried at RT [37].
4. Trypsin: Firstly, the nanoparticles were washed with Tris-HCl solution, then the mixture of trypsin (45 g/L) and Tris-HCl was added. The sample was mixed for 90 min at RT, and then for 30 min in 4 °C (ice bath). At the end, the solution was removed and the nanoparticles were washed with Tris-HCl three times and dried at RT [15–16,34,38].
